# Drug-Like Small Molecule HSP27 Functional Inhibitor Sensitizes Lung Cancer Cells to Gefitinib or Cisplatin by Inducing Altered Cross-Linked Hsp27 Dimers

**DOI:** 10.3390/pharmaceutics13050630

**Published:** 2021-04-28

**Authors:** Hawon Yoo, Seul-Ki Choi, Jaeok Lee, So Hyeon Park, You Na Park, Soo-Yeon Hwang, Jae-Ho Shin, Younghwa Na, Youngjoo Kwon, Hwa Jeong Lee, Yun-Sil Lee

**Affiliations:** 1 Graduate School of Pharmaceutical Sciences, Ewha Womans University, Seoul 120-720, Korea; ysv1060@hanmail.net (H.Y.); skchoi1028@ewhain.net (S.-K.C.); leejo19@ewha.ac.kr (J.L.); okp5310@naver.com (S.H.P.); youna211@naver.com (Y.N.P.); syhwang428@ewha.ac.kr (S.-Y.H.); 2 College of Pharmacy, CHA University, Pocheon 487-010, Korea; wogh975@naver.com (J.-H.S.); yna7315@cha.ac.kr (Y.N.)

**Keywords:** NSCLC, HSP27, heat shock protein, NA49, HSP27 inhibitor, drug resistance, EGFR, combination therapy

## Abstract

Relationships between heat shock protein 27 (HSP27) and cancer aggressiveness, metastasis, drug resistance, and poor patient outcomes in various cancer types including non-small cell lung cancer (NSCLC) were reported, and inhibition of HSP27 expression is suggested to be a possible strategy for cancer therapy. Unlike HSP90 or HSP70, HSP27 does not have an ATP-binding pocket, and no effective HSP27 inhibitors have been identified. Previously, NSCLC cancer cells were sensitized to radiation and chemotherapy when co-treated with small molecule HSP27 functional inhibitors such as zerumbone (ZER), SW15, and J2 that can induce abnormal cross-linked HSP27 dimer. In this study, cancer inhibition effects of NA49, a chromenone compound with better solubility, longer circulation time, and less toxicity than J2, were examined in combination with anticancer drugs such as cisplatin and gefitinib in NSCLC cell lines. When the cytotoxic drug cisplatin was treated in combination with NA49 in epidermal growth factor receptors (EGFRs) WT cell lines, sensitization was induced in an HSP27 expression-dependent manner. With gefitinib treatment, NA49 showed increased combination effects in both EGFR WT and Mut cell lines, also with HSP27 expression-dependent patterns. Moreover, NA49 induced sensitization in EGFR Mut cells with a secondary mutation of T790M when combined with gefitinib. Augmented tumor growth inhibition was shown with the combination of cisplatin or gefitinib and NA49 in nude mouse xenograft models. These results suggest the combination of HSP27 inhibitor NA49 and anticancer agents as a candidate for overcoming HSP27-mediated drug resistance in NSCLC patients.

## 1. Introduction

Heat shock proteins (HSPs) are evolutionally conserved molecular chaperones that assist the conformational protein folding or unfolding for performing their normal biological functions and are required for protein homeostasis and cell survival, protecting organisms from environmental stresses such as heat shock, irradiation, and chemical agents [1]. Of several HSPs including HSP70, HSP90, and HSP60, overexpression of small heat shock protein HSP27 (also known as HSPB1) is critical for cancer progression and metastasis and for the development of resistance to anticancer drugs [2]. HSP27 is frequently upregulated in the lung, breast, and pancreatic cancer cells and is involved in the cellular mechanisms of DNA repair, inhibition of apoptosis, and drug resistance [3,4,5]. Thus, HSP27 is a key combination therapy drug target for sensitizing drug-resistant cancer cells produced by chemotherapy [6,7]. In most non-small cell lung carcinoma (NSCLC) patients, epidermal growth factor receptors (EGFRs) are highly activated by exon19 deletion and L858R mutation or overexpression of the receptors [8,9]. An anticancer agent such as gefitinib, a tyrosine kinase inhibitor [10] inhibiting receptor tyrosine kinase activity of EGFR, shows good efficacy for patients with EGFR mutant lung cancers, but drug resistance and metastatic properties can occur [8,11]. One of the mechanisms leading to drug resistance is overexpression of HSP27, which occurs in various cancer patients [2,12,13]. Overexpression of HSP27 suppresses apoptotic cell death caused by diverse stimuli such as oxidative stress and γ-irradiation [14]. In addition, clinical trials have revealed a correlation between the level of HSP27 and aggressiveness of various cancers, metastatic properties, and the development of anticancer drug resistance, resulting in poor survival rates after chemotherapy and irradiation therapy [12,15,16]. Unlike other HSPs such as HSP70 and HSP90, which utilize ATPase activity for protein folding, HSP27 functions as a chaperone through the formation of large oligomers [17]. Although it is an appealing cancer target, HSP27 acts through an ATP-independent mechanism and is not susceptible to inhibition, unlike general small molecule-derived HSP70 or HSP90 inhibitors [18]. Small molecules such as quercetin and RP101 (also known as brivudine and bromovinyldeoxyuridine) can directly bind to the HSP27 protein, protein aptamers that can bind to the HSP27 protein, and the antisense oligonucleotide [19] that targets the mRNA that encodes HSP27 [20] have been analyzed. RP101 is a nucleoside that binds to Phe29 and Phe33 of HSP27, resulting in the inhibition of chemo-resistance and metastasis [21,22]. OGX-427, an HSP27 antisense oligonucleotide, acts to decrease the expression of HSP27 [23]. Zerumbone (ZER), a cytotoxic component isolated from the plant *Zingiber zerumbet Smith*, induced cross-linking of HSP27 protein in a time- and dose-dependent manner by insertion between the disulfide bonds of HSP27. Because ZER is a natural product-derived compound, large-scale production is limited. Moreover, the chemical characteristics of ZER hinder its use as a drug because of low solubility [24]. The chemical structure of a drug determines its physicochemical properties; affects its absorption, distribution, metabolism, excretion, and toxicity (ADME/Tox) properties; and ultimately affects its pharmacological activity. Research for drug-like properties aims to design compounds with potentially good ADME/Tox properties in the early phase of drug discovery. Accordingly, experiments were conducted using a potent HSP27 cross-linker SW15, which is a synthetic xanthone compound. SW15 induced sensitization to NSCLC cells in combination with taxol, cisplatin, HSP90 inhibitor (17-AAG), and radiation [25]. The chromenone compound J2, possessing better cross-linking formation activity on HSP27 than the xanthone compound SW15, was designed and synthesized by replacement of oxygen with nitrogen in the 4-pyron structure to overcome the limitation of quinolone compounds in medicine. We further identified that the combination of chemotherapy with J2, which forms altered cross-linking on HSP27, overcame resistance to anticancer drugs in HSP27-overexpressing cancer cells [26]. However, due to the poor solubility and in vivo circulation time of J2, we aimed to identify more druggable compounds with the same efficacy as J2. Of the compounds, NA49, a chromenone compound, was identified to show the desired effects. Similar to J2, NA49 can induce HSP27 cross-linking, abnormal dimerization, and combination effects in cancer cells. Moreover, the combination effects of NA49 with cisplatin or EGFR-TKI (gefitinib) were evaluated because these are popular treatments in NSCLC patients. Compared to J2, NA49 has lower toxicity and a superior pharmacokinetic profile. In addition, an animal study showed that NA49 can significantly inhibit lung cancer cell growth without observable toxicity. Based on these data, we suggest NA49 as a promising HSP27 functional inhibitor drug candidate for anticancer purposes of lung cancer cells in combination with anticancer modalities. In addition, our efforts to identify HSP27 inhibitors will support the development of approaches to target relatively unstructured regulatory proteins that have been designated as important but difficult targets.

## 2. Materials and Methods

### 2.1. Chemicals and Reagents

J2 derivatives including NA49 were chemically synthesized (Supplementary Figure S1). Acetonitrile was obtained from Merck (Darmstadt, Germany), and 0.9% normal saline was supplied by JW Pharmaceutical Corporation (Dangjin, Korea). Trifluoroacetic acid (TFA), *N*, *N*-dimethylacetamide (DMAc), and Tween^®^ 80 were purchased from Sigma-Aldrich (St. Louis, MO, USA). Gefitinib (ZD1839, #S1025) was purchased from Selleckchem, and cisplatin was purchased from Sigma Aldrich.

### 2.2. Animals for Pharmacokinetic and Toxicity Experiments

Male Sprague–Dawley rats (7-week-old) and male Institute of Cancer Research (ICR) mice (5-week-old) were purchased from Orient Bio (Seongnam, Korea) and Samtako Bio (Osan, Korea), respectively. The experimental animals were housed under conventional conditions and fasted overnight before surgery for catheterization. All procedures for animal experiments were in accordance with the guidelines by the Ewha Woman’s University Institutional Animal Care and Use Committee (IACUC, 15-049 and 16-062), Korea.

### 2.3. HPLC Analysis

The HPLC system used for this study was an Agilent HP1100 equipped with a quaternary pump with degasser, a variable wavelength detector, a thermostatted auto-sampler, and an HPLC 2D Agilent ChemStation software. A capcell-pak C_18_ MG120 column (3.0 × 250 mm, 5 µm, Shiseido, Tokyo, Japan) was used at 25 °C. The mobile phase was composed of water (0.1% trifluoroacetic acid) and acetonitrile (0.1% trifluoroacetic acid) (57:43 (*v*/*v*) for J2 and 50:50 (*v*/*v*) for NA49, respectively), filtered through a 0.45 μm Millipore filter and degassed prior to use, and the flow rate was 0.5 mL/min. UV detection was performed at 248 nm for J2 and at 246 nm for NA49, and the injection volume was 40 µL for both compounds. The analytical method was validated by FDA Bioanalytical Method Validation Guidance for Industry (U.S. Food and Drug Administration. Available online: https://www.fda.gov/regulatory-information/search-fda-guidance-documents/bioanalytical-method-validation-guidance-industry (accessed on 24 August 2020). To confirm the specificity of the analytical method, the chromatograms of rat plasma spiked with J2 and SW15 (internal standard (IS)) or NA49 and NA27 (IS) were compared with those of blank rat plasma. Calibration curves of J2 and NA49 in rat plasma were constructed at nine concentrations in the range of 0.025–10 µg/mL. The sensitivity of detection was evaluated by the lower limit of quantification (LLOQ), the drug concentration corresponding to the peak area that was 5-fold greater than the baseline noise. The intra-day precision and accuracy were estimated by analyzing five sets of four quality control (QC) samples (0.025, 0.05, 0.5, and 5 µg/mL) in the same day. The inter-day precision and accuracy were evaluated by analyzing a set of four QC samples on five days. The precision was expressed as a coefficient of variation (CV%) by calculating the ratio of standard deviation to mean of the measured drug concentration. The accuracy (%) was calculated by dividing the mean of the measured drug concentration by the theoretical drug concentration. The recovery of the analytes from plasma samples was evaluated at low and high concentrations (0.05 and 5 µg/mL, respectively) by comparing the peak areas of the analytes after extraction with those before extraction. Using low (0.5 µg/mL) and high (5 µg/mL) concentrations of the analytes, the short-term stability tests were conducted after standing for 4 h at room temperature for J2 and 6 h for NA49, while the long-term stability tests were performed after standing for 2 weeks and 4 weeks at −20 °C and −70 °C, respectively. The stability of the analytes following three freeze–thaw cycles, post-preparative stability, and stock solution stability also was examined. Each blood sample collected from the experimental animals was transferred into an Eppendorf tube and centrifuged at 13,000 rpm for 20 min. Each prepared plasma sample (50 µL) was mixed sequentially with 5 µL of IS solution and 45 µL of acetonitrile. After extraction, the supernatant was transferred to a clean insert for HPLC analysis.

### 2.4. Pharmacokinetic Study

J2 and NA49 were dissolved in 10% DMAc and 10% Tween^®^ 80 solution in saline to a concentration of 2.5 mg/mL for pharmacokinetic studies. The common carotid artery of the rat was catheterized under anesthesia using isoflurane the day before administration of J2 and NA49 [27]. Rats were divided into four groups. J2 and NA49 were administered to rats by intravenous (IV) injection at doses of 2 mg/kg and 10 mg/kg, respectively. Blood samples (0.2~0.25 mL) were collected at 0, 0.033, 0.083, 0.167, 0.33, 0.67, 1, 2, 3, 5, 7, 9, and 24 h following IV administration. The plasma samples obtained after centrifugation of the blood samples were stored at −20°C until HPLC analysis. The following pharmacokinetic parameters of J2 and NA49 were estimated by non-compartmental analysis using WinNonlin^®^ Professional version 5.2 software (Pharsight Corporation, Mountain View, CA, USA): Initial drug concentration (C_0_), area under the plasma concentration–time curve from 0 h to the final sampling time point (AUC_0-last_), elimination half-life (t_1/2_), apparent volume of distribution (V_d_), and total clearance (Cl).

### 2.5. Single-Dose Toxicity and Cytotoxicity 

For single-dose toxicity study of each drug, ICR mice were divided into 6 groups of control (0.9% normal saline), vehicle, and four dosages of each inhibitor (*n* = 4 mice per group). Both inhibitors dissolved in the same solvent system as the pharmacokinetic study were administered to the mice by caudal vein injection at doses of 2.5, 7.5, 15 and 30 mg/kg. After a single administration, all mice were observed daily for general conditions including behavior, hair, eyes, and nose. In addition, body weight was measured on days 0, 3, 7, and 14 following IV administration. 

For cytotoxicity analysis of J2 and NA49, cells were treated with a series of concentrations (0.01, 0.1, 1, 10, and 100 μM) over 24 h. The normal mammalian cells used were HFL-1: human embryonic lung cell line; L929: NCTC clone 929, mouse fibroblast cell line; NIH 3T3: mouse embryonic fibroblast cell line; CHO-K1: Chinese hamster ovary cell line; and VERO: African green monkey kidney cell line. To perform the Ames test of J2 and NA49, the number of revertant colonies was counted on each compound-treated plate at the maximum concentration at which the compound was soluble and nontoxic to the *S. typhimurium* tester strains (Supplementary Material S1). The ratio of the number of revertant colonies in the treated plate to colonies in the vehicle plate [2]. The values of revertant colonies per plate with [Factor] of positive controls were 462 ± 24 [28.9] for 2-nitrofluorene (2 μg/plate) against TA98 without S-9 mix; 415 ± 7 [24.4] for benzo(a)pyrene (2 μg/plate) against TA98 with S-9 mix; 441 ± 16 [4.1] for sodium azide (1 μg/plate) against TA98 without S-9 mix; and 852 ± 17 [6.3] for benzo(a)pyrene (2 μg/plate) against TA100 with S-9 mix. For the hERG K^+^ channel binding assay of J2 and NA49, the inhibitory activity against the hERG K^+^ channel and its ligand was measured using a red fluorescent hERG channel ligand tracer. The final activity was assessed as a decrease in the degree of fluorescence polarization.

### 2.6. Physicochemical Properties and In Vitro Metabolic Stability

Kinetic solubility (at pH 7) and logarithm of the partition coefficient (log P) of J2 and NA49 were determined through nephelometry and the pH-metric method, respectively. Permeability was evaluated with a parallel artificial membrane permeability (PAMPA) assay using an artificially generated lipid-infused membrane. In vitro metabolic stability of J2 and NA49 was assessed with liver microsomal phase I stability assay as the percentage of remaining parent compound after 30 min in the presence of mouse, rat, and human liver microsomes, respectively. In vitro human plasma stability of J2 and NA49 was evaluated as the percentage of remaining parent compound after 1 h treatment with human plasma. The effect of J2 and NA49 on CYP450 enzyme activity was tested at concentrations of 0.05~50 µM.

### 2.7. Cell Culture

The human NSCLC cell lines of NCI-H460, A549, HCC827, PC9, NCI-H1650, and NCI-H1975 were obtained from the American Type Culture Collection (Rockville, MD, USA). Cells were cultured in RPMI 1640 medium containing 10% FBS, 2 mmol/L L-glutamine, and 100 units/mL of penicillin and streptomycin and maintained at 37 °C in a humidified incubator containing 5% CO_2_.

### 2.8. Cell Transfection

HSP27 expression was suppressed using specific siRNAs of siHSP27 (sc-29350) and siControl (used as negative control, sc-37007), purchased from Santa Cruz Biotechnology. For transfection, cells were seeded in culture dishes, and transfection was performed after 24 h using Opti-MEM media (Invitrogen, Carlsbad, CA, USA) containing Lipofectamine 2000 reagent (Invitrogen). Lentiviruses were used to create stable NCI-H460 cell lines expressing shRNA for HSP27 (puromycin resistance gene). The HSP27 shRNA Plasmid (sc-2935-SH) and Transfection Reagent (sc-108061) for shRNA plasmid were ordered from Santa Cruz Biotechnology. To generate shControl and shHSP27 cells, the cell lines were transduced with 1 mol of lentivirus and selected using puromycin (1 µg/mL) for at least one week.

### 2.9. Viability Assay (3-(4,5-dimethylthiazol-2-yl)-2,5-diphenyltetrazolium Bromide (MTT) Assay)

Cell viability against gefitinib-, cisplatin-, and NA49-induced toxicity was determined using an MTT (Amersham Pharmacia Biotech) assay in 96-well plates. The NCI-H460, A549, HCC827, PC9, NCI-H1650, and NCI-H1975 cells were seeded at a density of 1 × 10^4^ cells/well in 96-well plates and treated with the desired concentration of gefitinib, cisplatin, or NA49 (at 0, 5, 10, 20, 40, 60, 80, 100, and 120 µM) for 24 h. Next, cells were incubated with 100 µL of 5 mg/mL 3-(4,5-dimethylthiazol-2-yl)-2,5-diphenyltetrazolium bromide (Sigma Aldrich, M5655) for 2 h. After the MTT mixture was removed, 100 µL of DMSO was added to each well, and absorbance at 540 nm was measured using an ELISA plate reader (TECAN, Twinfinite PRO). 

### 2.10. Western Blot

For polyacrylamide gel electrophoresis (PAGE) and Western blot (WB) analysis, cells were lysed with radioimmunoprecipitation assay (RIPA) buffer (50 mM Tris-HCl [pH 7.5], 150 mM NaCl, 1% NP-40, 0.1% sodium dodecyl sulfate (SDS), and 1% sodium deoxycholate) supplemented with 1 mM Na_3_VO_4_, 1 mM dithiothreitol (DTT), 1 mM phenyl-methyl-sulfonyl fluoride (PMSF), and protease inhibitor cocktail (Calbiochem). The samples were boiled for 5 min, and equal amounts of protein were analyzed by SDS-PAGE.

### 2.11. Antibodies

Goat polyclonal anti-HSP27 (sc-1049), mouse monoclonal anti-β-actin (sc-47778), and rabbit polyclonal anti-cyclin D1 (sc-753) antibodies were purchased from Santa Cruz Biotechnology. Rabbit polyclonal anti-cleaved caspase-3 (#9661), rabbit polyclonal anti-cleaved PARP (#9541), rabbit anti-EGFR (#2232), mouse monoclonal anti-phospho-EGFR (#2236), mouse monoclonal anti-HSP27 (#2402), and rabbit monoclonal anti-Bcl-2 (#3498) antibodies were purchased from Cell Signaling.

### 2.12. Flow Cytometry Analysis (FACS)

Cells were plated in 6-well culture plates at a density of 1 × 10^5^ cells/well. Compounds were applied to the cells immediately after seeding. When cells were harvested, cells were washed once with 1× phosphate-buffered saline (PBS), dissociated using Trypsin-EDTA, and centrifuged at 13,000 rpm for 3 min at 4 °C. Next, 1 mL of 1× PBS was added to wash the pellet again. Finally, 1 mL of 1× PBS and 1 μg/mL of propidium iodide (PI) were added to each polystyrene round-bottom tube. Flow cytometric analysis was performed using a FACS flow cytometer (BD Bioscience). To measure cell death, 10^4^ cells were counted, and PI-stained cells were analyzed. FACS analysis in accordance with the manufacturer’s instruction.

### 2.13. Tumor Xenografts in Mice

NCI-H460 single-cell suspension (1 × 10^6^ cells) was injected subcutaneously into the hind legs of 6-week-old BALB/c nude mice (Orientbio, Korea). When tumors reached a minimal volume of 150 mm^3^, xenograft mice were treated 7 times with J2 or NA49 by local regional application with or without treatment with cisplatin (2 mg/kg) by intraperitoneal (IP) injection. J2 or NA49 (20 mg/kg) was injected by IP every two days, and cisplatin was injected twice a week. NCI-H1650 single-cell suspension (1 × 10^7^ cells) was injected subcutaneously into the hind leg of 6-week-old NOD-SCID mice (Koatech, Korea). When tumors reached a minimal volume of 150 mm^3^, xenograft mice were treated 7 times with J2 or NA49 by local regional application with or without gefitinib (5 mg/kg) by IP injection. For this, J2 or NA49 (20 mg/kg) was injected by IP every two days, and gefitinib was injected every three days. Tumor volumes were determined according to the formula (L × l^2^)/2, by measuring tumor length (L) and width (l) with a caliper. Tumors were measured every other day and allowed to grow for 15 or 34 days. All procedures for animal experiments were in accordance with the guidelines by the Ewha Woman’s University Institutional Animal Care and Use Committee (IACUC, 18-006), Seoul, Korea.

### 2.14. Immunohistochemical Staining

Immunohistochemical staining of Ki-67, HSP27 (1:100; Cell Signaling #50353), and EGFR (1:100; Cell Signaling #4267) was performed on the most representative section of NCI-H460 and NCI-H1650 tumor tissue. Briefly, deparaffinization and hydration with xylene/ethanol at different concentrations (100%, 90%, 80%, and 70%) were performed. Next, the sections were treated for heat-mediated antigen retrieval using 10 mM citrate buffer (PH 6.0) for 30 min. Endogenous peroxidase activity was quenched by 3% hydrogen peroxide solution for 10 min. Non-specific binding was prevented by incubation with 5% normal goat serum for 1 h at 37 °C. After that, the sections were incubated with anti-human primary antibody for 3 h at 4 °C in a moist chamber. Secondary antibody incubation and staining were performed using the EnVision^®^ + System–HRP (ABC) kit (Vector Laboratories, Burlingame, CA, USA) according to the manufacturer’s recommendations and observed using light microscopy (Carl Zeiss, Oberkohen, German).

### 2.15. Statistics

Values are displayed as mean plus or minus SD or SEM. Comparisons between groups were carried out by one-way ANOVA for experiments with more than three sub groups. Post hoc range tests were performed with one-way ANOVA. Results were considered statistically significant for P values less than 0.05. Student’s *t*-test and *f*-test were performed for comparison of pharmacokinetic parameters between two dose groups of J2 or NA49.

## 3. Results

### 3.1. Synthesis of NA49 and J2 

**Figure 1:**
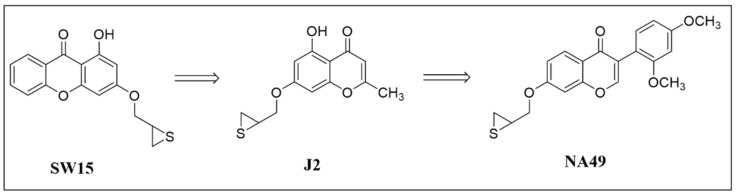
Structural changes of SW15, J2, and NA49. Structural changes of NA49, which is more druggable than SW15 and J2.

We previously reported that J2, a chromenone, is an anticancer compound that inhibits HSP27 chaperonic activity by acting as a crosslinker to form altered dimerization of HSP27 [26]. J2 derivatives including NA49 were chemically synthesized and confirmed by NMR and mass spectroscopic methods (Figure 1 and Supplementary Figure S1). We confirmed that NA49 formed altered dimerization of HSP27 with a very similar degree to that produced by the interaction of HSP27 with J2. The degree of NA49-induced apoptosis makers, cleaved PARP and cleaved caspase 7, was also similar to that of J2 (Supplementary Figure S2). This suggests that NA49 is HSP27-specific and non-toxic, as is J2 [25,26].

### 3.2. Characterization of Cytoxicity, Physicochemical Properties, and Metabolic Stability of NA49 and J2

To evaluate the druggable potential of J2 and NA49, cytotoxicity against normal mammalian cells, genotoxicity, and cardiac toxicity were evaluated (Table 1). Compounds with IC_50_ greater than 10 μM in a normal cell line were considered non-cytotoxic [28,29]. Since the IC_50_ values of J2 and NA49 are in the ranges of 91.8 μM to more than 100 μM and 83.6 μM to 96.5 μM, respectively, it is likely that these compounds are generally non-toxic. To evaluate the genotoxicity of the compounds, the Ames test was performed using the strains TA98 and TA100 of *Salmonella typhimurium*. Treatment of 40 μg/plate of J2 for 48 h increased bacterial reverse mutation in both TA98 and TA100 compared with the vehicle control, while treatment of 200 μg/plate of NA49 for 48 h showed no significant increase in a number of revertant colonies. 

Induction of a greater than two-fold increase in the number of revertant colonies compared to that of the vehicle control is judged as genotoxic at the treated concentration [30]. The possibilities of cardiac toxicities of J2 and NA49 were also evaluated with hERG K^+^ channel ligand binding assay and hERG patch-clamp assay (Table 1). J2 and NA49 inhibited hERG ligand binding to hERG K^+^ channel protein by 8.7% and 39.0%, respectively, while E-4031, a well-known experimental antiarrhythmic drug that blocks potassium channels of hERG [31], exhibited 99.6% inhibition. An inhibition rate of 50% or more by a compound applied at a concentration of 10 μM indicates that it participates in hERG K^+^ channel binding [32,33]. J2 and NA49 are thus not expected to cause inhibition of hERG K^+^ channel activity.

The physicochemical properties of J2 and NA49 were evaluated. The log P values of J2 and NA49 were 2.78 and 3.04, respectively, according to the pH-metric method. The kinetic solubility was 73.2 ± 1.1 μM (19.5 ± 0.3 μg/mL) for J2 and 90.2 ± 2.6 μM (33.4 ± 1.0 μg/mL) for NA49 using the nephelometry method. Cell permeability of J2 and NA49 was determined to be −4.90 ± 0.047 and −4.49 ± 0.152 using the PAMPA assay. Cell permeability of J2 is low, while that of NA49 is moderate (Table 2) [34]. The metabolic stabilities of J2 and NA49 were evaluated with in vitro microsomal stability assay and in vitro human plasma stability assay. Metabolic stability can affect pharmacokinetic parameters such as drug clearance, half-life, and oral bioavailability and is considered an important characteristic of drug candidates [35]. Though J2 and NA49 are likely to be relatively unstable in liver microsomal phase I, this might not persist in in vivo conditions [36,37]. We further evaluated in vitro plasma stability of J2 and NA49. The measured values for J2 were 76.2% and 82.7% when treated with 0.5 μg/mL and 5 μg/mL, respectively, and those for NA49 were 146.6% and 101.4%. This indicates that the half-life values of J2 and NA49 can be predicted to be about 3 h and longer than 6 h, respectively (Table 2) [38]. The differences in plasma stability and predicted half-life between J2 and NA49 can be attributed to the removal of a hydroxyl group at the C-5 position of the chromen-4-one ring in the metabolic pathway of NA49. The differences also might be due to the position of the substituent at C-3. To determine the exact reason for the higher stability of NA49 compared to J2, the further metabolic study is necessary.

Since drug–drug interactions can be predicted by measuring the degree of inhibition of CYP450 enzyme activity, the inhibitory activities of J2 and NA49 were measured against five CYP450 isotypes of 1A2, 2C9, 2C19, 2D6, and 3A4. These isotypes have been reported to play an important role in drug metabolism in humans [39,40]. J2 significantly inhibited CYP1A2 without affecting the activity of other CYP450 isotypes, whereas NA49 moderately inhibited only CYP2C9 (Table 3). Based on these results, when J2 is administered in combination with drugs metabolized by CYP1A2, such as α-naphthoflavone and acetaminophen, inhibition of J2 metabolism and increase in J2 blood concentration could be induced, leading to toxicity below the expected concentration of J2. Thus, in terms of drug–drug interaction, NA49 has fewer limitations of usage than J2.

### 3.3. Analytical method Validation and Pharmacokinetics of J2 and NA49

A simple HPLC-UV method was developed for the measurement of both J2 and NA49 in rat plasma. The peaks of J2 (retention time (RT), 9.6 min) and NA49 (RT, 20.0 min) were successfully eluted without endogenous interference. The equations for linearity ranged from 0.025 to 10 µg/mL and were as follows: y = 1.1029x + 0.0052 and y’ = 0.8976x’ + 0.0111, where y and y’ were the peak area ratios of J2 and NA49 to each IS, respectively, and x and x’ were the concentrations of J2 and NA49. The correlation coefficient (r^2^) was 1.00 for both inhibitors, and the LLOQ was 0.025 µg/mL for both. The intra- and inter-day accuracy of J2 and NA49 varied from 88.1 to 112.2% and 93.8 to 106.5%, respectively. The CVs of intra- and inter-day precisions of J2 and NA49 were between 1.2 and 8.7% and between 3.7 and 11.2%, respectively.

All the values for accuracy and precision were within ± 15%, which is the acceptable range. The mean values of extraction recovery for J2 and NA49 were 96.5 ± 1.18% (low) and 110.8 ± 3.39% (high) and 103.8 ± 3.93% (low) and 95.4 ± 1.80% (high), respectively, and the mean recovery values of SW15 and NA27 (ISs for J2 and NA49) at the working concentration were 97.6 ± 6.58% and 106.4 ± 3.77%. The stability test results for short-term, long-term, and freeze–thaw storage of J2 were between 87.1 and 116.6%, while those of NA49 were between 86.7 and 105.6%. Moreover, the stability of standard stock solution (1 mg/mL) and a post-preparative sample of J2 were 113.2% and 102.2–103.4%, respectively, while those of NA49 were 98.7% and 96.9–102.4%. The stability results suggested that NA49 was stable (less than ± 15%) at the conditions required for sample storage and handling. On the other hand, J2 was stable except at −70°C for 4 weeks and three freeze–thaw cycles at low concentration (0.05 µg/mL), which showed means of 115.2% and 116.6%, respectively (greater than ±15%).

**Figure 2:**
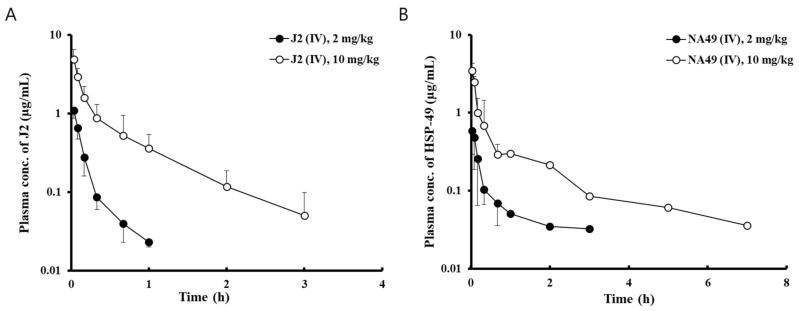
Mean plasma concentration–time profiles of J2 and NA49. (**A**) J2 and (**B**) NA49 were intravenously injected into rats. Bars represent S.D. (*n* = 5–9). (●) 2 mg/kg; (○) 10 mg/kg.

Figure 2 showed mean plasma concentration after IV administration of J2 and NA49 for the indicated times at two doses (2 mg/kg and 10 mg/kg) in rats. Table 4 represent the pharmacokinetic parameters of J2 and NA49. The mean values of C_0_ and AUC_0-last_ of J2 were increased in a dose-dependent manner, while the mean values of V_d_ and Cl were not changed with increasing dose. Based on pharmacokinetic parameters and mean plasma concentration *versus* time profile, J2 was eliminated from the body very quickly, having a short elimination half-life compared to NA49 (Figure 2). The mean values of C_0_ and AUC_0-last_ of NA49 at a dose of 10 mg/kg increased 4.8-fold and 5.2-fold, respectively, compared to those at a dose of 2 mg/kg, while the mean values of t_1/2_, V_d_, and Cl were similar at the two doses, suggesting linear kinetics (Table 4).

### 3.4. Single-Dose Toxicity of J2 and NA49

Four doses of 2.5, 7.5, 15, and 30 mg/kg of J2 and NA49 were administrated to ICR mice by a single tail vein injection. The maximum dose for the two inhibitors was 30 mg/kg because of the limit of solubility. To evaluate the toxicity of J2 and NA49, mouse activity, injury, and body weight were monitored and recorded for 14 days following IV administration. All mice survived to the final day except for one mouse in the group of 30 mg/kg of NA49 (Table 5). The LD_50_ value was not achieved by 30 mg/kg of the two inhibitors following IV injection. Mean body weight increased in a time-dependent manner for all groups (data not shown). There were no significant changes in body weight of all study groups compared to control and vehicle groups. In addition, notable differences were not detected in the activities and appearances of all study groups.

### 3.5. NA49 Showed Similar HSP27 Cross-Linking Activity to J2

To accurately compare HSP27 cross-linking activity between NA49 and J2, Western blotting for HSP27 was compared using HSP27 recombinant protein. Altered dimerization of HSP27 by both J2 and NA49 was observed, and NA49 showed stronger cross-linking activity than J2. Pretreatment with N-acetyl-L-cysteine (NAC) reduced J2- or NA49-mediated cross-linked HSP27 dimers, as NAC was more likely to form reversible disulfide bonds with HSP27, thereby interfering with the ability of J2 or NA49 to form covalent bonds with HSP27. This suggested that the cysteine residue of HSP27 is important for J2- or NA49-mediated cross-linking of HSP27 (Supplementary Figure S3A) [24]. When J2 or NA49 at an indicated concentration was applied to an NCI-H460 lung cancer cell line, altered dimerization was detected, and cross-linking activity of NA49 was slightly less than J2, which might be due to cellular permeability (Table 2 and Supplementary Figure S3B).

### 3.6. Cytotoxicity of Cisplatin, Gefitinib, and NA49 in EGFR WT and Mut Lung Cancer Cell Lines

To identify the characteristics of lung cancer cells, six NSCLC lines were compared: NCI-H460 and A549 are EGFR wild type (WT) cell lines; PC9 (exon 19 deletion) and HCC827 (exon 19 deletion) are EGFR-mutated (Mut), gefitinib (Gef)-sensitive cells. NCI-H1650 (exon 19 deletion) and NCI-H1975 (L858R and T790M) are EGFR-mutated (Mut), Gef-resistant cells. EGFR Mut cells showed higher expression of HSP27 than did EGFR WT cells. Moreover, EGFR Mut Gef-resistant cells showed higher expression of HSP27 than did EGFR Mut Gef-sensitive cells (Supplementary Figure S4 and Table 6).

To reveal whether HSP27 crosslinker shows sensitization to cisplatin-mediated cell death in an HSP27 expression-dependent manner, EGFR WT, NCI-H460, and A549 cell lines were treated with cisplatin (0, 3, 5, and 10 µM) or NA49 (0, 5, 10, and 20 µM), and Western blotting was performed.

**Figure 3:**
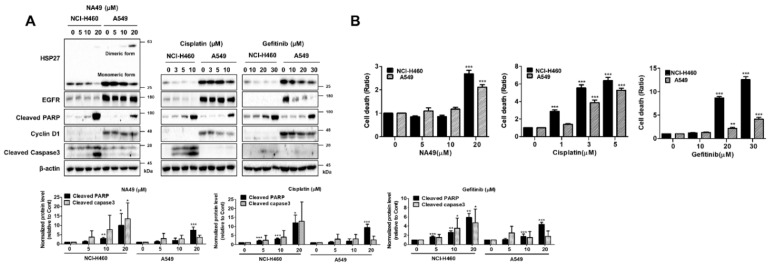
HSP27 cross-linking activities of NA49 and effect of cisplatin or gefitinib in EGFR wild-type lung cancer cell lines. (**A**) NCI-H460 and A549 cells were treated with NA49 (0, 5, 10, and 20 μM), cisplatin (0, 3, 5, and 10 μM), or gefitinib (0, 10, 20, and 30 μM) for 24 h, and cell lysates were detected by Western blot analysis. Protein levels were quantified using Image J software. The data were expressed as the fold change relative to the control and normalized to β-actin in graph. (**B**) NCI-H460 and A549 cells were treated with NA49 (0, 5, 10, and 20 μM), cisplatin (0, 3, 5, and 10 μM), or gefitinib (0, 10, 20, and 30 μM) for 48 h, and cell death was analyzed by flow cytometry after treatment with propidium iodide (PI). Results are the mean and standard deviation of three independent experiments (* *p* < 0.05, ** *p* < 0.01, *** *p* < 0.001 vs. untreated control cells.).

The results showed no effect of NA49 on apoptosis marker expression or cell death at doses less than 20 µM. However, for 20 µM of NA49, higher apoptosis in was noted in NCI-H460 than A549 cells, and NA49 sensitivity was higher in NCI-H460 than A549 cells. Similarly, cisplatin showed greater cell death in NCI-H460 than A549 cells even at lower doses. Gef also showed higher sensitivity in NCI-H460 than A549 cells even though EGFR activation was greater in A549 than NCI-H460. Moreover, Gef sensitivity was well correlated with HSP27 expression level (A549 showed higher expression of HSP27 than did NCI-H460 cells). Therefore, in EGFR-WT cells, sensitivity to NA49, cisplatin, and Gef was more highly correlated to HSP27 than to EGFR expression level (A549 showed both higher HSP27 and EGFR expression than did NCI-H460 cells) (Table 6 and Figure 3).

**Figure 4:**
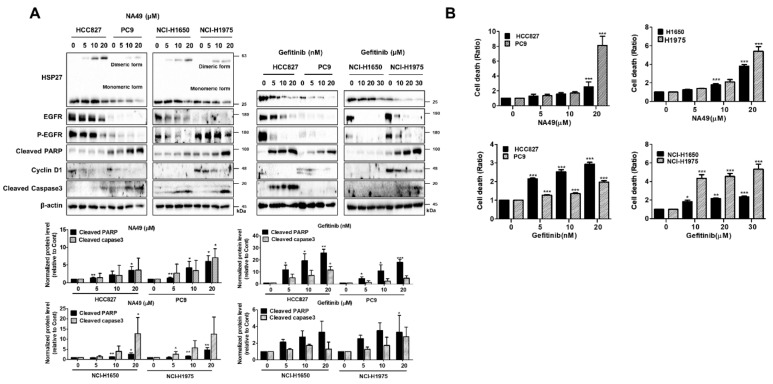
HSP27 cross-linking activities of NA49 and effect of gefitinib in EGFR mutant-type lung cancer cell lines. (**A**) NCI-H1650 and NCI-H1975 cells were treated with NA49 (0, 5, 10, and 20 μM) or gefitinib (0, 10, 20, and 30 μM) for 24 h, and cell lysates were detected by Western blot analysis. Protein levels were quantified using Image J software. The data were expressed as the fold change relative to the control and normalized to β-actin in graph. (**B**) NCI-H1650 and NCI-H1975 cells were treated with NA49 (0, 5, 10, and 20 μM) or gefitinib (0, 10, 20, and 30 μM) for 48 h, and cell death was analyzed by flow cytometry after propidium iodide (PI) staining. Results are the mean and standard deviation of three independent experiments (* *p* < 0.05, ** *p* < 0.01, *** *p* < 0.001 vs. untreated control cells).

In EGFR Mut cell line, PC9 and H1975 showed high sensitivity of NA49 than HCC827 and H1650. Increased cell death due to Gef even at nM level concentrations was shown in EGFR Mut Gef-sensitive cell lines HCC827 and PC9 (HCC827 was more sensitive to Gef than was PC9). In contrast, EGFR Mut Gef-resistant cell lines NCI-H1650 and NCI-H1975 showed resistance to Gef (NCI-H1975 was more sensitive to Gef than NCI-H1650). Gef sensitivity was dependent on EGFR activation level in both Gef-sensitive and –resistant EGFR Mut cells but was not dependent on HSP27 expression level (Figure 4A,B). IC_50_ values for cisplatin and NA49 are summarized in Table 6.

### 3.7. NA49 Showed Sensitization Effects in Combination with Cisplatin or Gefitinib in Both EGFR Wild Type and Mutant Lung Cancer Cell Lines

Cisplatin is a potent anticancer drug, especially in EGFR WT tumors. However, resistance can emerge early due to compensatory mechanisms involving increased expression of HSP27 [10], which inhibits drug effectiveness. Gef is used for the treatment of lung cancer with EGFR WT [41].

**Figure 5:**
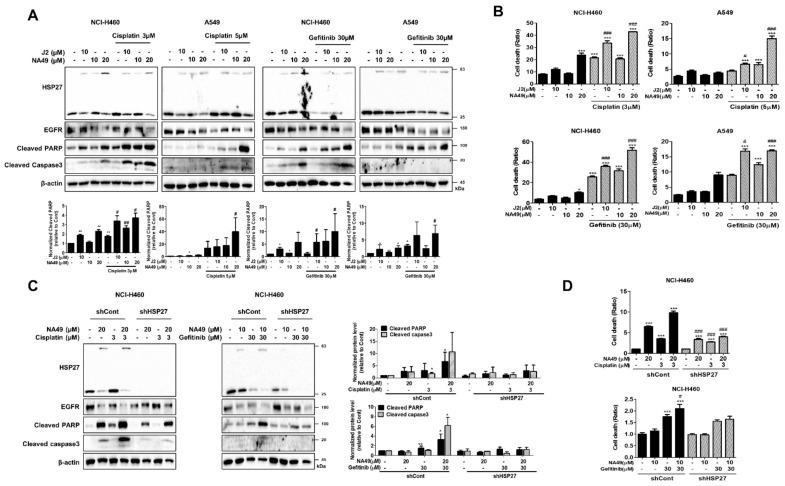
HSP27 cross-linking activities of J2 and NA49 in combination with cisplatin or gefitinib in EGFR wild-type lung cancer cell lines. (**A**) NCI-H460 and A549 cells were treated with J2 (10 μM) or NA49 (10 or 20 μM) for 24 h, with or without cisplatin (3 μM) or gefitinib (30 μM), and cell lysates were detected by Western blot analysis. Protein levels were quantified using Image J software. The data were expressed as the fold change relative to the control and normalized to β-actin in the graph. (**B**) NCI-H460 and A549 cells were treated with J2 (10 μM) or NA49 (10 or 20 μM) for 48 h, with or without cisplatin (3 μM) or gefitinib (30 μM), and cell death was analyzed by flow cytometry after propidium iodide (PI) staining. Results are the mean and standard deviation of three independent experiments (* *p* < 0.05, ** *p* < 0.01, *** *p* < 0.001 vs. untreated-control; # *p* < 0.05, ## *p* < 0.01, ### *p* < 0.001 vs. cisplatin or gefitinib alone). (**C**) NCI-H460 cells stably transfected with control (shControl) or shRNA of HSP27 (shHSP27) were treated with NA49 (10 μM) with or without cisplatin (3 μM) or gefitinib (30 μM), and cell lysates were detected by Western blot analysis. Protein levels were quantified using Image J software. The data were expressed as the fold change relative to the control and normalized to β-actin in the graph. (**D**) Cell death was analyzed by flow cytometry after propidium iodide (PI) staining. Results are the mean and standard deviation of three independent experiments (* *p* < 0.05, ** *p* < 0.01, *** *p* < 0.001 vs. sh-control; # *p* < 0.05, ## *p* < 0.01, ### *p* < 0.001 vs. sh-HSP27).

To elucidate whether cross-linking activity of NA49 is correlated with sensitization effects, we compared J2 and NA49 in EGFR WT cells in combination with cisplatin or Gef. Based on the IC_50_ values, the dose of cisplatin for induction of better sensitization was 3 µM, and that of Gef was 30 µM, which showed less than 30% cell death in NCI-H460 and A549 cells. The effects of combination with cisplatin were more potent in J2- than NA49-co-treated cells at 10 µM and were dramatically increased when NA49 concentration was increased to 20 µM. Cleaved PARP and cleaved caspase-3 data suggested that co-treatment of J2 or NA49 with cisplatin synergistically sensitized the NCI-H460 and A549 lung cancer cells at 24 h (Figure 5A). PI staining for total cell death detection also suggested that J2 or NA49 potentially increased the cancer cell death in combination with cisplatin at 48 h. Treatment with 10 µM of J2 showed a greater sensitization effect in combination with cisplatin than did NA49, while 20 µM of NA49 dramatically induced sensitization. Gef co-treatment showed similar patterns of cisplatin co-treatment (Figure 5B). When combination index (CI) values were calculated, synergism effects of NA49 in combination with cisplatin or Gef was observed (Supplementary Table S1). The combination effects of NA49 with cisplatin or Gef were more strongly induced in A549 cells than NCI-H460 cells, which might be mediated by a higher level of HSP27. Moreover, the combination effects of NA49 were disappeared in cells with disruption of HSP27 cross-linking activity by stably transfection of shRNA of HSP27 (Figure 5C,D).

### 3.8. NA49 Showed Sensitization Effects in Combination with Gefitinib in EGFR Mutant Cells

**Figure 6:**
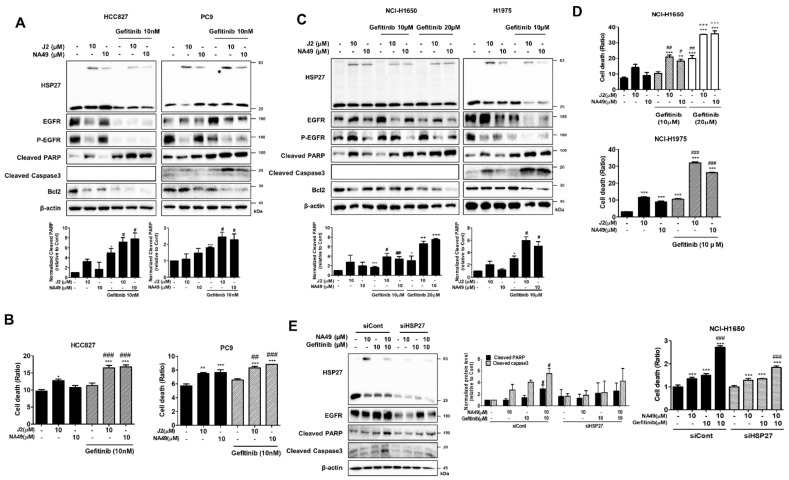
J2 and NA49 showed sensitization effects on EGFR mutant-type lung cancer cells in combination with gefitinib. (**A**) HCC827 and PC9 cells were treated with J2 (10 μM) and NA49 (10 μM) for 24 h, with or without gefitinib (10 nM), and cell lysates were detected by Western blot analysis. Protein levels were quantified using Image J software. The data were expressed as the fold change relative to the control and normalized to β-actin in the graph. (**B**) Cell death was analyzed by flow cytometry after propidium iodide (PI) staining. Results are the mean and standard deviation of three independent experiments (* *p* < 0.05, ** *p* < 0.01, *** *p*< 0.001 vs. untreated-control; # *p* < 0.05, ## *p* < 0.01, ### *p*< 0.001 vs. gefitinib alone). (**C**) NCI-H1650 cells were treated with J2 (10 μM) or NA49 (10 μM) for 24 h, with or without gefitinib (10 or 20 μM) and NCI-H1975 cells were treated with J2 (10 μM) or NA49 (10 μM) for 24 h, with or without gefitinib (10 μM), and cell lysates were detected by Western blot. Protein levels were quantified using Image J software. The data were expressed as the fold change relative to the control and normalized to β-actin in the graph. (**D**) Cell death was analyzed by flow cytometry after propidium iodide (PI) staining. Results are the mean and standard deviation of three independent experiments (* *p* < 0.05, ** *p* < 0.01, *** *p* < 0.001 vs. untreated-control; # *p* < 0.05, ## *p* < 0.01, ### *p* < 0.001 vs. gefitinib alone 10 μM; + *p* < 0.05, ++ *p* < 0.01, +++ *p* < 0.001 vs. gefitinib alone 20 μM). (**E**) NCI-H1650 cells transfected with control (siCont) or siRNA of HSP27 (siHSP27) were treated with NA49 (10 μM) with or without gefitinib (10 μM), cell lysates were detected by Western blot analysis, and cell death was analyzed by flow cytometry after propidium iodide (PI) staining. Protein levels were quantified using Image J software. The data were expressed as the fold change relative to the control and normalized to β-actin in the graph. Results are the mean and standard deviation of three independent experiments (* *p* < 0.05, ** *p* < 0.01, *** *p* < 0.001 vs. untreated-control; # *p* < 0.05, ## *p* < 0.01, ### *p* < 0.001 vs. gefitinib alone).

Gef is an EGFR-targeted, first-generation TKI drug used as an EGFR inhibitor. However, resistance emerges early due to the occurrence of other mutations, such as T790M (substitutes a threonine (T) with a methionine (M) at position 790 of exon 20) or other compensatory signaling activation. [42,43,44]. Gef concentrations that induced less than 50% cell death were selected (Table 6). When Gef (10 nM) was combined with J2 or NA49 in HCC827 and PC9 cells, with Gef sensitivity and apoptosis markers of cleaved caspase-3 and cleaved PARP were increased (Figure 6A).

In EGFR Mut cells, 10 μM of NA49 showed sensitization effects similar to those of J2. Cell death as detected with PI staining suggested that J2 or NA49 increased cell death in combination with Gef (Figure 6B). EGFR Mut Gef-resistant cell lines, such as NCI-H1650 and NCI-H1975 showed higher expression of HSP27 compared to other cell lines which were used in this study.

EGFR Mut Gef-resistant cells showed resistance to EGFR-TKI, requiring a relatively high dose of Gef. NCI-H1650 and NCI-H1975 cells showed potentiated combination effects when detected with apoptosis markers cleaved caspase-3 and cleaved PARP, suggesting that combination of J2 or NA49 with Gef sensitized lung cancer cells (Figure 6C). In addition, when cell death was detected using PI staining and CI was calculated, NA49 showed synergistically increased cell death by combined treatment with Gef, except for PC cells (PC9 cells induced enough cell death even at low doses of Gef alone) (Figure 6D and Supplementary Table S1). Similarly, sensitizing effects of NA49 were absent when shRNA of HSP27 was stably transfected (Figure 6E).

### 3.9. NA49 in Combination with Cisplatin or Gefitinib Sensitized Lung Cancer Cells in a Xenograft Mouse Model 

**Figure 7:**
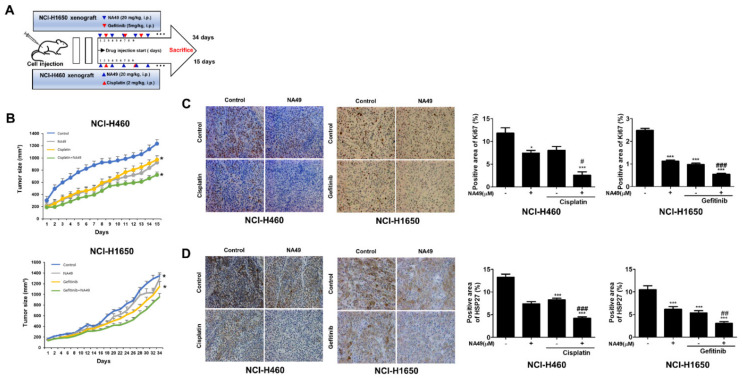
NA49 sensitized tumors in an NCI-H460 and NCI-H1650 cells-injected xenograft mouse model with cisplatin and ge-fitinib. (**A**) NCI-H460 and NCI-H1650 cells were injected subcutaneously into BALB/c nude mice (*n* = 6/group). Xenografted mice were treated 7 times with NA49 (20 mg/kg) intraperitoneal treatment in combination with four injections of cisplatin (2 mg/kg) or gefitinib (5 mg/kg). (**B**) Tumor size was measured every other day. Results are the mean and standard error. (**C**) Ki67 staining (**D**) and immunohistochemistry of HSP27 were performed using tumor tissues. Results are the mean and standard error (*p < 0.05, *** *p* < 0.001 vs. untreated-control; # *p* < 0.05, ## *p* < 0.01, ### *p* < 0.001 ### vs. cisplatin or gefitinib alone).

To confirm the in vitro results of the combination effect of NA49 with anticancer drug, in vivo xenograft studies were performed by implanting NCI-H460 cells (EGFR WT cells) or NCI-H1650 (EGFR Mut Gef-resistant cells) cells subcutaneously into the hind legs of mice. When tumors reached the size of 150 mm^3^, NA49 (20 mg/kg, IP, every other day) was injected with cisplatin (2 mg/kg, IP, twice a week) or Gef (5 mg/kg, IP, twice a week), and subsequent tumor growth was monitored (Figure 7A). 

A greater reduction in tumor growth in NCI-H460 cells- or NCI-H1650 cells-xenografted mice was detected in combination groups with NA49 compared to cisplatin or Gef alone (Figure 7B). Cell proliferation in tumor tissues was detected by Ki67 immunohistochemistry and correlated with the sensitizing effect of NA49 in combination with cisplatin or Gef (Figure 7C). To elucidate whether NA49 affects HSP27 expression, HSP27 immunohistochemistry was performed and showed that NA49, cisplatin, or Gef alone inhibited HSP27 expression and the combination showed greater synergistic inhibition (Figure 7D). Moreover, NA49 decreased EGFR expression to a greater extent than did Gef, suggesting that functional inhibition of HSP27 affects EGFR stability, even though more detailed experiments should be carried out to support this conclusion (Supplementary Figure S5).

## 4. Discussion

In this study, we suggested NA49, a small molecule following J2 with a similar structure of a chromenone compound, forms a similar level of altered cross-linking of HSP27 when compared to J2, but is more druggable form than J2 in better toxicity, IV pharmacokinetic, physicochemical properties, genotoxicity and metabolic stability. Because overexpression of HSP27 induced cellular resistance to anticancer drugs cisplatin and Gef, combination therapy with NA49 in NSCLC was performed. Combination of NA49 with Gef in EGFR-WT and Mut cells or with cisplatin in EGFR-WT cells synergistically induced combination effects to NSCLC cell lines, suggesting NA49 as a universal combination treatment candidate to combat HSP27-mediated resistance. Moreover, cross-linking of HSP27 by NA49 was specific for HSP27 protein, as demonstrated by loss of cross-linking activity in the shHSP27 cell line. In vivo data revealed anti-tumor activity of NA49 with cisplatin in EGFR-WT cells or with Gef in EGFR-Mut cells, indicating NA49 as an adjuvant candidate for anticancer therapy. Moreover, NA49 showed better druggability than J2, suggesting NA49 as a possible druggable candidate for HSP27 inhibition.

According to recent studies, ZER [24], isolated from a natural product, and SW15, a synthetic xanthone compound, induced cross-linking of HSP27 protein. They formed a covalent bond between the cysteine-thiol group of HSP27 and resulted in abnormal dimerization [25]. The side chains of the xanthone moiety affected the type of HSP27 cross-linking activity. The Cys residue of HSP27 is necessary for altered cross-linking of HSP27 by the xanthone compound J2, a synthetic chromenone compound with a pharmacophore structure and more potent cross-linking activity than SW15 [26]. However, the druggability of J2 is limited by toxicity, IV pharmacokinetic and physicochemical properties. Therefore, NA49 with the same chromenone structure as J2 was synthesized. Although the only difference between J2 and NA49 is an additional aromatic phenyl residue and loss of a hydroxyl group, druggability was better for NA49 than J2.

Although targeted therapy provides novel treatment options, chemotherapy remains the standard of care for most patients with NSCLC without driver mutations. Platinum-based chemotherapy like cisplatin is the treatment of choice for patients with adenocarcinoma [45]. In addition, progression-free survival (PFS) for platinum-based chemotherapy alone is approximately six months. Although several recent trials have demonstrated that immunotherapy combined with platinum-based chemotherapy can prolong PFS [46], the benefit for overall survival remains unclear. Therefore, novel treatment modalities are warranted. HSP27 expression was reported to involve chemotherapy resistance, and our results also suggest that a combination of cisplatin with HSP27 cross-linkers produced synergistic sensitization to NSCLC in EGFR-WT.

In our study, HSP27 expression was higher in EGFR-Mut cells than in EGFR-WT cells, which might be mediated by HSP27 phosphorylation by activated p38 MAPK. Among EGFR-Mut cells, Gef-resistant cells showed higher HSP27 levels than Gef-sensitive cells did, suggesting that HSP27 expression is well correlated with EGFR activation. Indeed, EGFR Mut Gef resistant cells showed increased phosphorylation of HSP27 than EGFR Mut Gef sensitive cells (unpublished data). 

EGFR-TKIs are effective first-line drugs in patients with EGFR-Mut cells. However, several studies have revealed a benefit in patients with EGFR-WT [30], even though the mechanism of which is not clear. Additionally, patients with EGFR-WT are primarily resistant to Gef [47,48]. NA49 augmented the cell death in EGFR-WT cells when combined with Gef, and depletion of HSP27 diminished these effects, suggesting HSP27 inhibition as a potential chemo-sensitization target for Gef treatment in EGFR-WT cancer cells. EGFR-TKIs have become first-line therapy drugs for patients with NSCLC harboring EGFR-Mut cells; the majority of patients with an initial response to Gef will develop resistance due to the effects of drug-resistant mutations through T790M mutations or other compensatory activation mechanisms [42,43,44]. Increased sensitization was observed in the combination of Gef with NA49 in EGFR-Mut Gef-resistant cells, which was well correlated with HSP27 expression level. Moreover, NA49 also showed sensitization in Gef-resistant NSCLC cells with secondary mutation of EGFR such as T790M, suggesting that HSP27 inhibition can overcome Gef resistance, which is mediated by diverse pathways. 

HSP27 strongly modulates drug resistance and suggests a potential target for anticancer treatment, especially for overcoming EGFR-mediated resistance. However, to make a more clear conclusion, further studies on the relationship between EGFR and HSP27, especially the role of HSP27 in EGFT-TKI-induced resistance development, should be performed.

## 5. Conclusions

In conclusion, HSP27 is frequently overexpressed in NSCLC and is associated with the development of resistance against anticancer drugs. Therefore, inhibitors of HSP27 can improve cancer chemotherapy when used in combination with anticancer drugs. In this sense, small molecules of cross-linking HSP27 are promising for functional inhibition of HSP27 and druggable NA49 may be a good candidate.

## Figures and Tables

**Table 1:** Toxicity of J2 and NA49.

Compound				J2	NA49
IC_50_ (μM) of compounds in various normal mammalian cells *^a^*		HFL-1		>100	94.5
		L929		>100	91.9
		NIH3T3		>100	96.5
		CHO-K1		>100	90.4
		VERO		91.8	83.6
Ames test	Amount treated (μg/plate)	Tester strain	TA98	40	200
			TA100	40	200
	Revertant colonies/plate *^b^* (Mean ± S.D.) [Factor] ^#^	Without S-9 mix	TA98	57 ± 7 [3.6]	19 ± 3 [1.2]
			TA100	643 ± 70 [6.0]	229 ± 2 [2.0]
		With S-9 mix	TA98	65 ± 2 [3.8]	22 ± 2 [1.3]
			TA100	418 ± 9 [3.1]	262 ± 11 [1.9]
hERG K^+^ Channel Binding Assay *^c^*		Concentration treated (μM)		10	10
		% Inhibition of hERG ligand binding		8.7 ± 2.22	39.0 ± 3.9

*^a^* Cells were treated for 24 h in a series of concentrations (0.01, 0.1, 1, 10, and 100 μM). *^b^* Evaluating the number of revertant colonies of compound-treated plate. The test was conducted with maximum concentration at which the compound was soluble and nontoxic to the *S. typhimurium* tester strains. ^#^ Factor = No. of revertant colonies of treated plate/colonies of vehicle control plate. *^c^* Measuring the inhibitory activity against hERG K^+^ channel and its ligand using a red fluorescent hERG channel ligand tracer. Data are presented as mean ± S.D.

**Table 2:** Physicochemical properties and stability of J2 and NA49.

Compound		J2	NA49
Solubility (pH 7.0) *^a^*		73.2 ± 1.1 μM (19.5 ± 0.3 μg/mL)	90.2 ± 2.6 μM (33.4 ± 1.0 μg/mL)
Log P *^a^*		2.78	3.04
Cell Permeability *^b^* Log *P*_app_ (cm/s)		−4.90 ± 0.047	−4.49 ± 0.152
In vitro Metabolic Stability *^c^* (% of remaining after 30 min)	Mouse	6.2 ± 2.55	2.4 ± 0.95
	Rat	2.6 ± 0.45	5.0 ± 1.33
	Human	47.8 ± 4.50	24.9 ± 0.77
In vitro Human Plasma Stability at 37 °C (% of remaining after 1 h)	When treated with 0.5 μg/mL	76.2 ± 0.72	146.6 ± 0.01
	When treated with 5 μg/mL	82.7 ± 0.73	101.4 ± 0.13

*^a^* Kinetic solubility at pH 7 and log P was determined through nephelometry and the pH-metric method, respectively. *^b^* Permeability was evaluated with a PAMPA assay. *^c^* In vitro metabolic stability was assessed with liver microsomal phase I stability assay. Data are presented as mean ± S.D.

**Table 3:** IC_50_ (μM) values *^a^* of J2 and NA49 against isotypes of CYP450 enzyme.

Isotype of CYP450 /Compound	J2	NA49
1A2	0.1	>50
2C9	>50	19.2
2C19	>50	>50
2D6	>50	>50
3A4	>50	>50

*^a^* CYP450 enzyme activity was measured with treatment of each compound at the concentrations of 0.05~50 μM. The positive controls used for each isotype are acetaminophen for 1A2, OH-tolbutamide for 2C9, OH-omeprazole for 2C19, dextrophan for 2D6, and OH-midazolam for 3A4. When treated with 10 μM of each positive control, the % inhibition values were 92.4 (acetaminophen), 91.7 (OH-tolbutamide), 93.2 (OH-omeprazole), 94.0 (dextrophan), and 92.5 (OH-midazolam).

**Table 4:** Mean pharmacokinetic parameters following IV injection in rats.

Pharmacokinetic Parameters	Drug	2 mg/kg	10 mg/kg
C_0_ (µg/mL)	J2	1.64 ± 0.73	6.96 ± 2.60 ***
	NA49	0.72 ± 0.40	4.96 ± 1.12 ***
AUC_0–last_ (µg·h/mL)	J2	0.18 ± 0.05	1.48 ± 0.75 ***
	NA49	0.21 ± 0.07	1.24 ± 0.59 ***
t_1/2_ (h)	J2	0.19 ± 0.07	0.45 ± 0.30 *
	NA49	1.40 ± 0.84	1.55 ± 0.48
V_d_ (L)	J2	0.77 ± 0.27	1.05 ± 0.72
	NA49	3.35 ± 1.69	4.13 ± 1.87
Cl (L/h)	J2	2.79 ± 0.69	1.90 ± 0.76
	NA49	1.84 ± 0.49	1.80 ± 0.56

* *p* < 0.05 and *** *p* < 0.001 compared with 2 mg/kg group; Data are presented as mean ± S.D. (*n* = 5–9).

**Table 5:** Survival rate of ICR mice after a single IV administration of J2 and NA49 (*n* = 4).

Group/Compound	Survival Rate (%)	
	J2	NA49
Control	100	100
Vehicle	100	100
2.5 mg/kg	100	100
7.5 mg/kg	100	100
15 mg/kg	100	100
30 mg/kg	100	75

**Table 6:** Characterization of non-small cell lung cancer (NSCLC) cell lines.

Cell Lines	EGFR	EGFR Expression	HSP27 Expression	Cisplatin IC_50_ (µM)	NA49 IC_50_ (µM)
NCI-H460	Wild-type	+	+	11.7	38.05 ± 0.69
A549	Wild-type	+++	++	14.4	62.48 ± 0.90
HCC827	Mutant-type (Exon19 del)	+++++	+++		64.42 ± 2.31
PC9	Mutant-type (Exon19 del)	+++	+++		72.99 ± 2.66
NCI-H1650	Mutant-type (Exon19 del)	++	+++++		62.94 ± 1.58
NCI-H1975	Mutant-type (L858R, T790M)	+++	++++		89.44 ± 2.57

## Data Availability

Not applicable.

## Supplementary Materials

The following are available online at https://www.mdpi.com/article/10.3390/pharmaceutics13050630/s1, Figure S1: Synthetic method and NMR spectrum of NA48-54, Figure S2: HSP27 cross-linking and apoptosis-inducing activities of J2 derivatives including NA49, Figure S3: NA49 showed HSP27 cross-linking activity the same as that of J2, Figure S4: Expression levels of EGFR and phosphorylated EGFR in lung cancer cell lines, Figure S5: EGFR expression in tumor of NCI-H1650 xenograft mouse model, Table S1: CI values of NSCLC cell lines, Supplementary Material S1: HPLC Analysis and Ames test.

## Abbreviations

Non Small Cell Lung Cancer (NSCLC), Heat Shock Protein (HSP), Heat Shock Protein 27 (HSP27), Epidermal Growth Factor Receptor (EGFR), Epidermal Growth Factor Receptor-Tyrosine Kinase Inhibitor (EGFR-TKI), Quality Control (QC), Coefficient of Variation (CV), Parallel Artificial Membrane Permeability Assay (PAMPA), Lower Limit of Quantification (LLOQ), Retention Time (RT), N-acetyl-L-cysteine (NAC), Standard Deviation (SD), Standard error of the mean (SEM).
