# Supplementary material for: Drug-Like Small Molecule HSP27 Functional Inhibitor Sensitizes Lung Cancer Cells to Gefitinib or Cisplatin by Inducing Altered Cross-Linked Hsp27 Dimers
Source: Pharmaceutics. 2021 Apr 28;13(5):630. doi: 10.3390/pharmaceutics13050630 (PMC8145107; doi:10.3390/pharmaceutics13050630)
Supplement: Supplementary file 1 [file pharmaceutics-13-00630-s001.zip › pharmaceutics-1167186-SI.pdf]

pharmaceutics

MDPI

Supplementary Materials: Drug-like Small Molecule HSP27 Functional Inhibitor Sensitizes Lung Cancer Cells to Gefitinib or Cisplatin by Inducing Altered Cross-linked Hsp27 Dimers

Hawon Yoo, Seul-Ki Choi, Jaeok Lee, So Hyeon Park, You Na Park, Soo-Yeon Hwang, Jae-Ho Shin, Younghwa Na, Youngjoo Kwon, Hwa Jeong Lee and Yun-Sil Lee

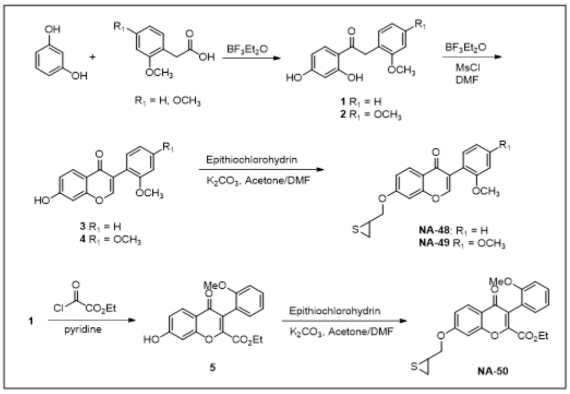

Scheme 1. Synthetic method for **NA 48-50**

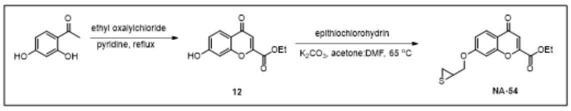

Scheme 4. Synthetic method for **NA 54**

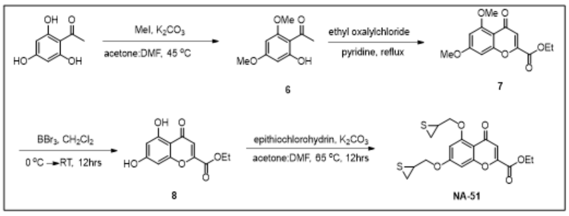

Scheme 2. Synthetic method for **NA 51**

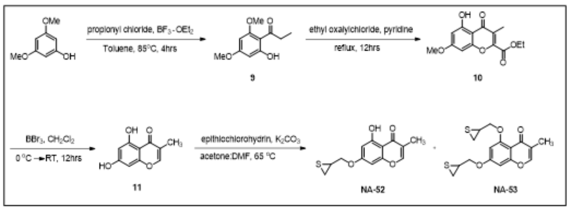

Scheme 3. Synthetic method for **NA 52** and **53**

^{1}H NMR Spectrum of NA48

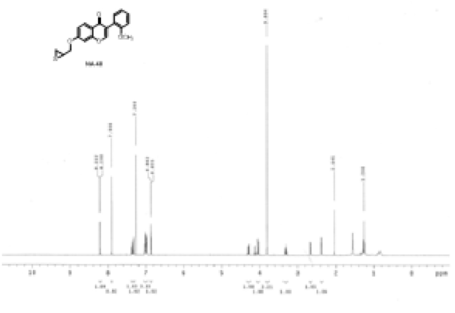

^{13}C NMR Spectrum of NA48

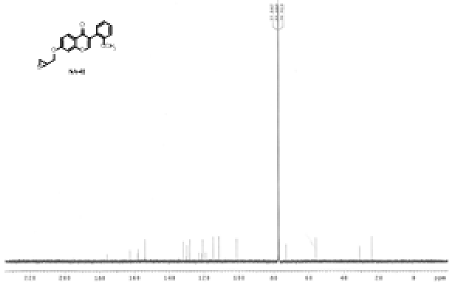

^{1}H NMR Spectrum of NA49

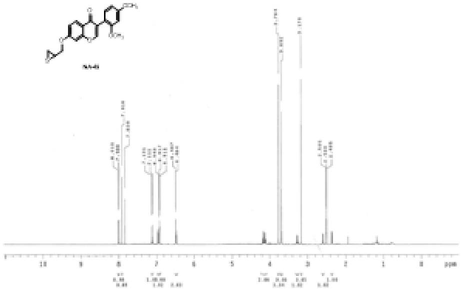

^{13}C NMR Spectrum of NA49

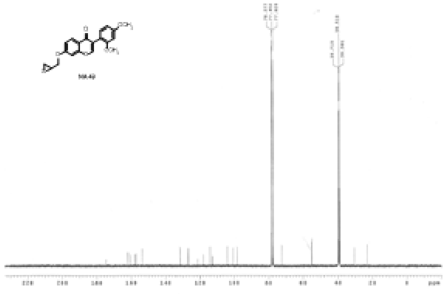

^{13}C NMR Spectrum of NA50

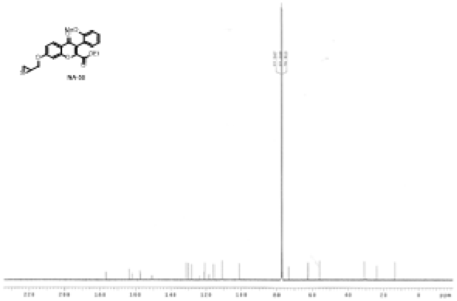

^{1}H NMR Spectrum of NA51

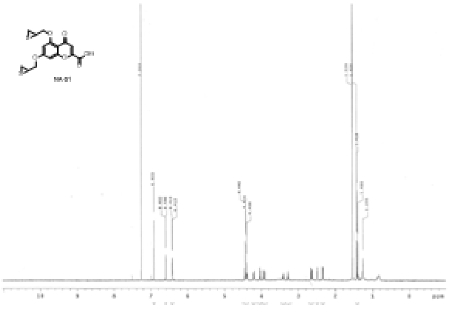

^{13}C NMR Spectrum of NA51

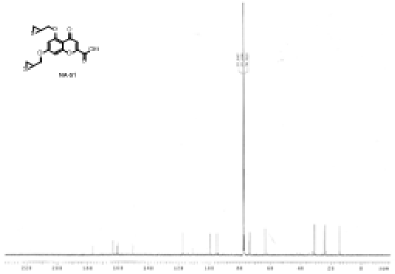

^{1}H NMR Spectrum of NA48

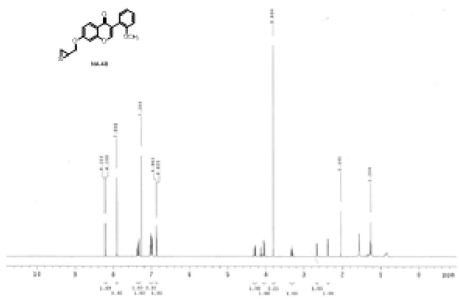

^{13}C NMR Spectrum of NA48

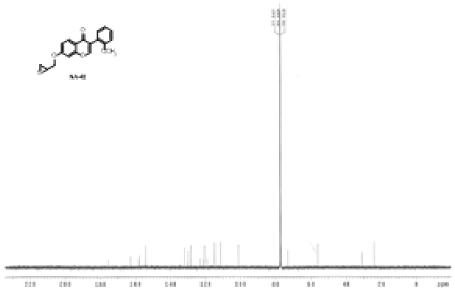

^{1}H NMR Spectrum of NA49

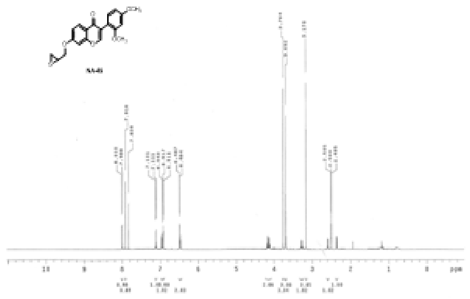

^{13}C NMR Spectrum of NA49

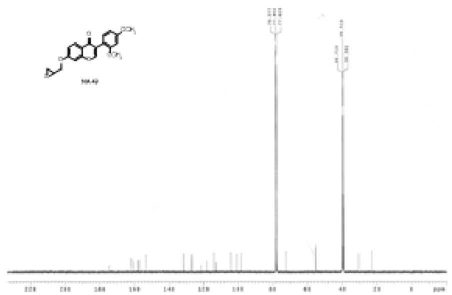

^{13}C NMR Spectrum of NA50

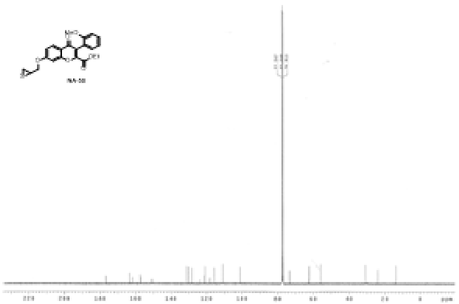

^{1}H NMR Spectrum of NA51

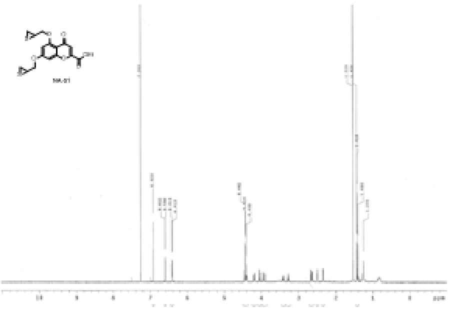

^{13}C NMR Spectrum of NA51

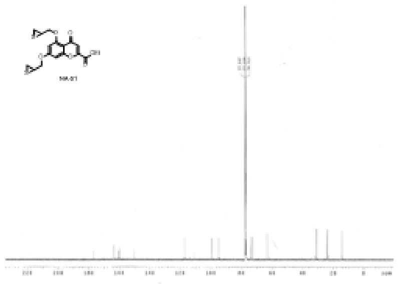

Figure S1. Synthetic method and NMR spectrum of NA48–54.

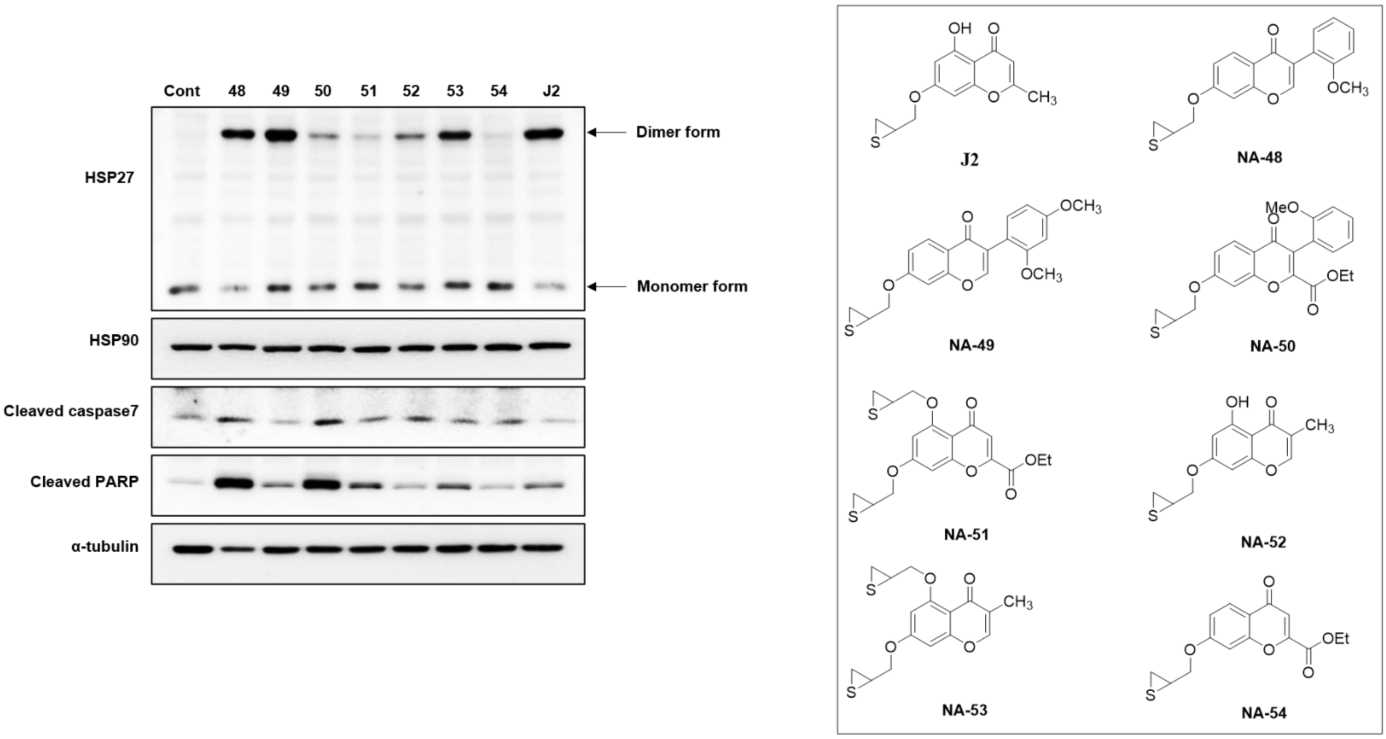

Figure S2. HSP27 cross-linking and apoptosis-inducing activities of J2 derivatives including NA49. (**A**) NCI-H460 cells were treated with NA48-54 (10 μM) and J2 (10 μM) for 24 h, and cell lysates were detected by Western blot analysis. (**B**) Structures of compounds J2 and NA48-54.

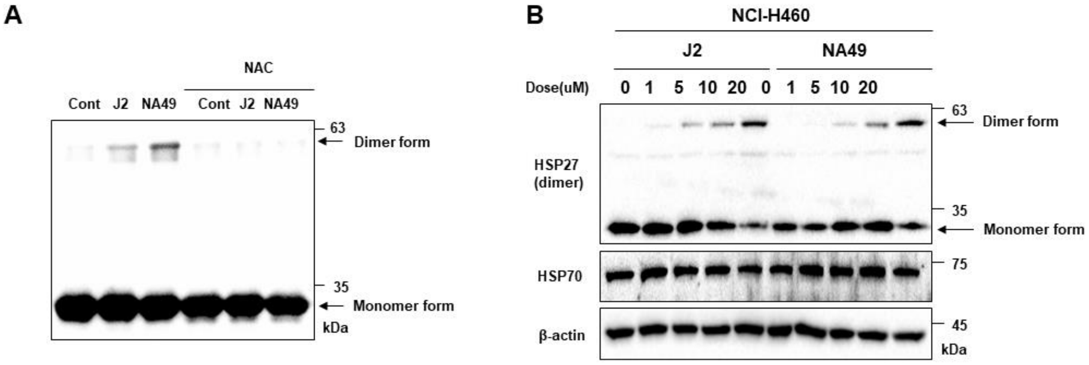

Figure S3. NA49 showed HSP27 cross-linking activity the same as that of J2. (**A**) HSP25 WT protein was pretreated with NAC (*N*-acetyl-L-cysteine) at 20 mM for 30 min and then treated with J2 or NA49 at 10 μM. After 3 h, the dimerization of proteins was altered. (**B**) NCI-H460 cells were treated with J2 or NA49 (0, 1, 5, 10 and 20 μM) for 24 h, and cell lysates were detected by Western blot analysis.

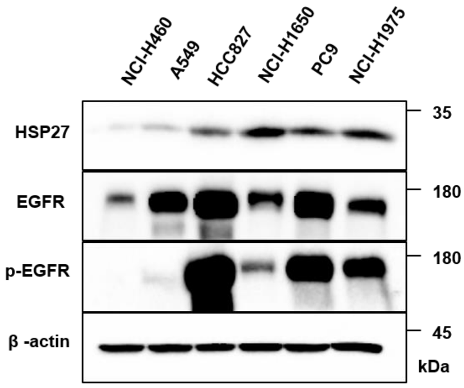

Figure S4. Expression levels of EGFR and phosphorylated EGFR in lung cancer cell lines. Each cell line was incubated after 24 h, and cell lysates were detected by Western blot analysis.

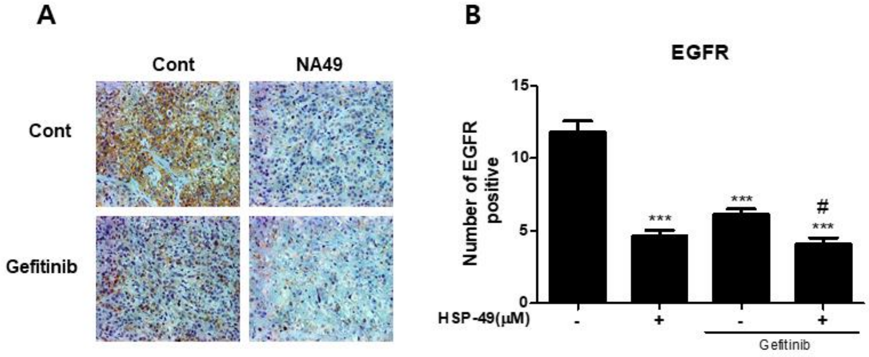

Figure S5. EGFR expression in tumor of NCI-H1650 xenograft mouse model. (**A**) Immunohistochemistry of EGFR was performed using tumor tissues. (**B**) Quantification represented EGFR expression. Graph represents mean and standard deviation (*$*p < 0.05$, ***$p < 0.001$ vs. control and #$p < 0.05$ vs. cisplatin alone group).

Table S1. CI values of NSCLC cell lines.

Cell line	Combination drug	NA 10 μM	NA 20 μM
	**Cisplatin**		
H460	3 μM	0.942	0.969
A549	5 μM	0.698	0.823
	**Gefitinib**		
HCC827	0.01 μM	0.651	
PC9	0.01 μM	1.84	
H1650	10 μM	0.828	
	20 μM	0.487	
H1975	10 μM	0.392	

CI values were calculated from the data of percent inhibition of cell death by PI staining. Each CI value was analyzed by CompuSyn software.

Supplementary material S1. Materials and Methods

HPLC Analysis

The HPLC system used for this study was an Agilent HP1100 series system, which consisted of a model 1100 quaternary pump with degasser pump, a model 1100 variable wavelength detector, a model 1100 thermostatted auto-sampler and a model HPLC 2D Agilent ChemStation software. A capcell-pak C_{18} MG120 column (3.0 × 250 mm, 5 μm, Shiseido, Tokyo, Japan) was used at 25 °C. The mobile phase was composed of water (0.1% trifluoroacetic acid) and acetonitrile (0.1% trifluoroacetic acid) (57:43 (*v*/*v*) for J2 and 50:50 (*v*/*v*) for NA49, respectively), filtered through a 0.45 um Millipore filter and degassed prior to use, and the flow rate was 0.5 mL/min. UV detection was performed at 248 nm for J2

and 246 nm for NA49, respectively, and the injection volume was 40 μL for both compounds. The analytical method of the analytes was validated by FDA Bioanalytical Method Validation Guidance for Industry. The chromatogram of the rat plasma spiked with J2 and SW15 (IS) or NA49 and NA27 was compared with that of the blank rat plasma in order to confirm the specificity of the analytical method. The calibration curves of J2 and NA49 in rat plasma was built for nine concentration levels in the range of 0.025–10 μg/mL. The sensitivity of detection was evaluated by the lower limit of quantification (LLOQ), the drug concentration corresponding to the peak area that was 5-fold greater than the baseline noise. The intra-day precision and accuracy were estimated by analyzing five sets of four quality control (QC) samples (0.025, 0.05, 0.5 and 5 μg/mL) in the same day. The inter-day precision and accuracy were evaluated by analyzing a set of four QC samples on five different days. The precision was expressed as a coefficient of variation (CV%) by calculating the ratio of the standard deviation to the mean measured drug concentration. The accuracy (%) was calculated by dividing a mean measured drug concentration by the theoretical drug concentration. The recovery of the analytes from the plasma samples was evaluated at low and high concentrations (0.05 and 5 μg/mL, respectively) by comparing the peak areas of the analytes after extraction to those before extraction. For the stability tests, low (0.5 μg/mL) and high (5 μg/mL) concentrations of the analytes were used. The short-term stability tests were conducted at room temperature for 4 h for J2 and 6 h for HSP49, while the long-term stability tests were performed at −20°C and −70°C for 2 weeks and 4 weeks, respectively. The stability of the analytes following three freeze-thaw cycles, the post-preparative stability and the stock solution stability were also examined. Each blood sample collected from the experimental animals was transferred into an Eppendorf tube and centrifuged at 13,000 rpm for 20 min. The prepared plasma sample (50 μL) was mixed with 5 μL of IS solution and 45 μL of acetonitrile, sequentially. After extraction, the supernatant was transfer to clean insert for the HPLC analysis.

Ames test

The test was conducted with maximum concentration at which the compound was soluble and nontoxic to the *S. typhimurium* tester strains. [#]Factor = No. of revertant colonies of treated plate colonies of vehicle control plate. The values of revertant colonies/plate [Factor] of positive controls are 462 ± 24 [28.9] for 2-nitrofluorene (2 μg/plate) against TA98 without S-9 mix; 415 ± 7 [24.4] for benzo(a)pyrene (2 μg/plate) against TA98 with S-9 mix; 441 ± 16 [4.1] for sodium azide (1 μg/plate) against TA98 without S-9 mix; 852 ± 17 [6.3] for benzo(a)pyrene (2 μg/plate) against TA100 with S-9 mix. For hERG K^+ channel binding assay, measuring the inhibitory activity against the hERG K^+ channel and its ligand using red fluorescent hERG channel ligand tracer. Final activity was assessed by the decrease in fluorescence polarization degree.
